# Delayed Effect of Superheated Steam Treatment on Starch Retrogradation of Rice Cake After Storage by Modifying Starch Chain-Length Distribution in Rice Flour

**DOI:** 10.3390/molecules29225253

**Published:** 2024-11-06

**Authors:** Ruge Cao, Zixiu Zhang, Xishuang Wang, Chen Xia, Yongqiang Cheng, Zhiwei Wang, Ju Qiu

**Affiliations:** 1 Key Laboratory of Precision Nutrition and Food Quality, Department of Nutrition and Health, China Agricultural University, No. 17 Tsinghua East Road, Haidian District, Beijing 100083, China; xia453853384@163.com (C.X.); chengyq@cau.edu.cn (Y.C.); 2 State Key Laboratory of Food Nutrition and Safety, College of Food Science and Engineering, Tianjin University of Science and Technology, Tianjin 300457, China; rgcao@tust.edu.cn (R.C.); 22845989@mail.tust.edu.cn (Z.Z.); w13053655282@163.com (X.W.); wangzw@tust.edu.cn (Z.W.)

**Keywords:** superheated steam, pasting property, rice starch retrogradation, storage

## Abstract

This study investigated the effects of superheated steam (SS) treatment on the physicochemical properties of rice flour and the subsequent impact on rice cake quality. The SS-180 resulted in higher final viscosity and significantly increased pasting time and the temperature of rice flour (*p* < 0.05). A significant enhancement in the water holding capacity of rice flour (*p* < 0.05) was due to the rice starch aggregated in this SS process. SS treatment also led to an increase in the proportion of short chains of amylopectin in rice flour from 30.40% to 37.59%, while a decrease in long chains retarded retrogradation and improved rice cake texture. The hardness of the SS-treated rice cake was lower than that of the untreated one, but the specific volume was increased significantly (*p* < 0.05). After 7 days of storage, rice cake with SS-180 treatment showed the lowest hardness, namely, the lowest retrograded process. These findings underscore the potential of SS treatment to enhance the physicochemical properties of rice flour and improve rice cake quality.

## 1. Introduction

The demand for gluten-free alternatives is increasing due to the rising awareness of gluten intolerance and celiac disease, which affects about 1% of the global population [1]. Rice, a staple gluten-free carbohydrate, is a popular choice due to its digestibility, low allergenicity, and nutritional value, containing approximately 75% carbohydrates, 7%–8% protein, and 1.3%–1.8% fat, along with essential vitamins [2]. However, the use of native rice flour in products like rice cake is limited by its low shear resistance and high susceptibility to starch degradation [3]. These drawbacks contribute to deteriorated cake quality during storage, manifesting as increased hardness, reduced elasticity, water loss, and starch recrystallization [4]. While chemical additives such as anti-aging agents and emulsifiers can improve rice flour functionality, they pose environmental and safety concerns [5]. Therefore, physical modification techniques, especially non-chemical methods like superheated steam (SS) treatment, are gaining traction in enhancing rice flour properties.

SS treatment, an emerging thermal technology, involves heating saturated steam above its saturation point. It offers distinct advantages, including high heat transfer rates, reduced chemical dependency, and shorter processing times [6]. Compared to conventional heat treatments, SS treatment has demonstrated superior performance in maintaining the physicochemical integrity and nutritional quality of grains [7,8]. Additionally, studies have shown that SS can modify starch structure, enhance functional properties, and improve the physical characteristics of lightly polished rice [9,10]. In rice flour, SS treatment was reported to increase gelatinization levels and reduce pasting viscosities [11] while also enhancing starch interactions and gel stability [12,13]. These structural modifications influence the arrangement of amylose and amylopectin, which are crucial for starch functionality [14]. SS treatment was discovered to enhance wheat cake quality because of the modifications of amylose and amylopectin arrangement [15]. However, it is not clear whether this modification of rice starch induced by SS is definitely benefited for the rice cake quality during storage or not. Further research is necessary to fully understand the effects of SS treatment on rice flour properties and the corresponding cake quality.

Although existing studies have examined the effects of SS treatment on the characteristics of rice and wheat products, gaps remain in understanding how SS treatment alters amylopectin structure in rice flour, how these changes translate into rice cake quality, and how they impact starch retrogradation during storage. Previous work has mostly focused on the effects of SS on whole grains or flours for conventional applications, with limited exploration of its specific effects on rice flour used in gluten-free rice cakes.

This study aims to investigate the effects of SS treatment on the physicochemical properties of rice flour and its subsequent impact on rice cake quality. It is the first time illustrating the final effects of SS treatment on the storage quality of gluten-free rice cake. Specifically, this study focuses on the specific volume, texture, and storage stability of gluten-free rice cake, so as to clarify its relations with the changes in starch structure and physicochemical properties of rice flour induced by SS treatment with different temperature. This research provides valuable insights for the development of high-quality rice-based products, addressing key challenges in gluten-free food innovation.

## 2. Results

### 2.1. Effects of SS Treatment on the Composition and Particle Size Distribution of Rice Flour

**Figure 1:**
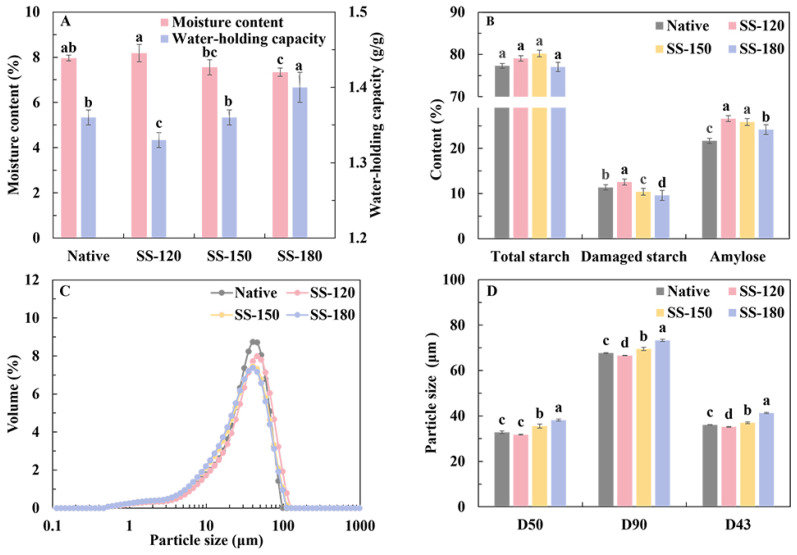
The composition and particle size distribution of rice flour: Moisture content and water holding capacity (**A**); total starch, damaged starch, and amylose content (**B**); particle size distribution (**C**); and average particle size (**D**). Native: untreated rice flour; SS-120: rice flour with superheated steam at 120 °C; SS-150: rice flour with superheated steam at 150 °C; SS-180: rice flour with superheated steam at 180 °C. Values with different superscript letters were significantly different, *p* < 0.05.

As shown in Figure 1A, SS-120 and SS-150 treatments retained the moisture content of rice flour, likely due to the formation of condensed water [16]. The moisture content of SS-180 treated rice decreased significantly (*p* < 0.05), due to the high temperature promoting the rapid evaporation of water inside the rice. The water holding capacity of rice flour, shown in Figure 1A, was significantly improved by the SS-180 treatment (*p* < 0.05). This improvement might be attributed to the expansion of starch particles caused by the high temperature.

In Figure 1B, the DS content of both native and SS-treated rice flour is presented. Notably, SS-120 treatment significantly increased the DS content (*p* < 0.05), while SS-150 and SS-180 treatments significantly decreased DS levels (*p* < 0.05). The increase in DS levels with the SS-120 treatment might be attributed to the presence of condensed water on the rice surface, which increased grain moisture and subsequently made the milling process more challenging, leading to increased starch damage during milling [16]. Conversely, SS-150 and SS-180 treatments significantly decreased DS levels (*p* < 0.05). This reduction might be due to the high-temperature SS treatment making the rice grains’ structure less dense and more prone to breakage, resulting in less mechanical damage to the starch during milling. Similar findings were reported by Hu et al. [16]. Additionally, starch–protein interactions might also reduce the content of damaged starch, a result consistent with previous research by Wang et al. [17]. Furthermore, research has shown that fine flours generally have a higher content of DS compared to coarse flours [18].

Figure 1B illustrates that the amylose content of SS-treated rice flour overall exceeds that of native rice flour, and the amylose content of SS-180 was significantly lower than that of SS-120 and SS-150 (*p* < 0.05). This phenomenon can be attributed to the breakdown of starch chains and the consequent reduction in its double helix structure beyond SS treatment, facilitating amylose dissolution, consistent with previous findings [9]. However, the amylose content in SS-180 rice flour was lower than that in SS-120 and SS-150 rice flour. This result might be due to excessive heat exposure leading to the degradation of starch molecules or the binding of amylose-amylose or amylose-amylopectin chains [19].

The particle size reduction in rice flour followed a unimodal pattern with peak concentration, as depicted in Figure 1C. Initially, the grain size of rice flour decreased but then increased as the SS temperature rose. The increase in particle size of SS-150 and SS-180 (*p* < 0.05) was attributed to the SS-treated rice becoming fluffy due to protein–starch aggregation, starch gelatinization, and structural expansion, which was proven by the morphological observation below. However, with the SS-120 treatment, the rice structure might become denser and harder due to condensed water (Figure 1A), making the milling process more challenging and resulting in decreased particle size [16]. These observations were further supported by D50, D90, and D43 values (Figure 1D).

### 2.2. Effect of SS Treatment on Rheological Properties of Rice Flour and Rice Paste

**Figure 2:**
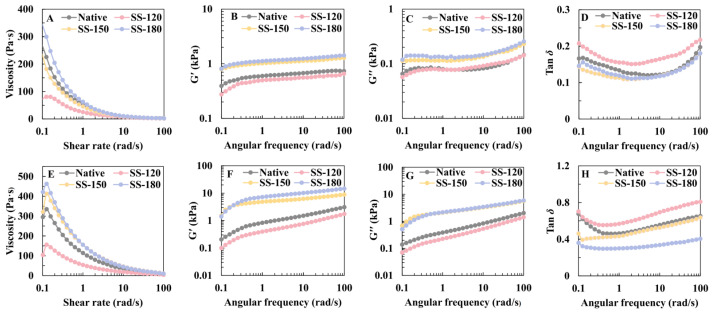
The rheological properties of rice flour and rice paste: Apparent viscosity of rice flour (**A**), variation in G’ (storage modulus) (**B**), G′′ (loss modulus) (**C**), and tan δ (loss tangent) (**D**). Apparent viscosity of rice paste (**E**), variation in G’ (storage modulus) (**F**), G′′ (loss modulus) (**G**) and tan δ (loss tangent) (**H**). Native: untreated rice flour; SS-120: rice flour with superheated steam at 120 °C; SS-150: rice flour with superheated steam at 150 °C; and SS-180: rice flour with superheated steam at 180 °C.

The rheological properties of rice flour and rice paste significantly impact cake quality. The rheology of rice flour is depicted in Figure 2A–D, while the rice paste is illustrated in Figure 2E–H. The apparent viscosity of SS-180 rice flour increased significantly, contrary to SS-120 and SS-150 rice flour, which showed a decrease (Figure 2A). Figure 2B,C presents the dynamic G’ and G′′ properties of the gel analyzed using angular frequency sweep tests. Both G’ and G′′ increased with angular frequency. As the angular frequency increased, the increase in G’ and G′′ was relatively gentle. Among the samples, the G’ and G′′ of SS-150 and SS-180 were very close and significantly improved the elasticity and viscosity of rice flour. This result aligned with the findings of Ma et al. [20]. The tan δ values of rice flour in Figure 1D suggested a “solid-like” behavior of rice starch gels, indicating dominant elastic behavior [9]. SS-120 increased tan δ, which was not beneficial for the rheological properties of rice flour. Conversely, the tan δ of SS-150 and SS-180 initially decreased and then aligned closely with that of untreated rice flour, indicating that SS-150 and SS-180 improved the rheological properties of rice flour to some extent.

Additionally, the rheological properties of the rice paste were measured. The results showed that SS-150 and SS-180 increased the apparent viscosity of rice paste, while SS-120 reduced it. This difference might be attributed to the high moisture content of SS-120 (Figure 1A), resulting in a thinner rice paste. Figure 2F,G displays the G’ and G′′ of rice paste, respectively. Their changes align with those of rice flour, but the G’ and G′′ of rice paste increase more rapidly with rising angular frequency. This outcome could be due to the interaction between other ingredients in the rice paste and rice flour, leading to a faster increase in viscoelasticity. Lastly, the tan δ of SS-180 rice paste decreased significantly, indicating that SS-180 improved the elastic properties of rice paste. In summary, the SS-180 batter exhibited better viscoelasticity, suggesting improved molecular segment entanglement and a strengthened gel network structure.

### 2.3. Effect of SS Treatment on Pasting Properties of Rice Flour

In Table 1, the peak viscosity (PV) of SS-treated flour was lower than that of native rice flour, likely due to starch degradation under SS treatment leading to smaller molecular chains [10]. SS-120 treatment reduced the PV the most, followed by SS-150 and SS-180, indicating that, as the temperature increased, the disorder of starch molecules increased, resulting in greater starch swelling [21].

SS-120 and SS-150 treatments decreased the breakdown of rice flour, indicating that these starch granules were more resistant to disintegration under continuous shear, thereby improving the thermal stability of the rice flour. Among these, SS-120 rice flour showed the lowest breakdown value and the best thermal stability [6]. The final viscosity (FV) of SS-120 significantly decreased, while the FV of others did not show significant changes. The pasting temperature of SS-treated rice flour was significantly higher than that of native rice flour, likely due to denaturation protein barriers surrounding the starch granules, which caused granule swelling at higher temperatures [22].

### 2.4. Amylopectin Chain-Length Distribution and Weight-Average Molecular Weight of Rice Starch

**Figure 3:**
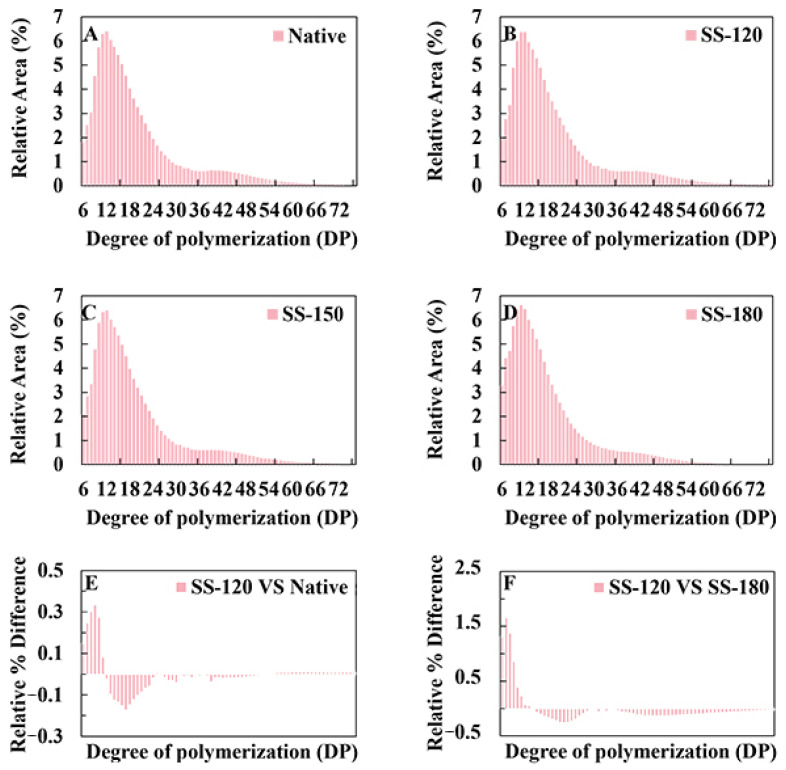
Amylopectin branch chain-length of rice starch. Untreated rice starch (**A**); rice starch with superheated steam at 120 °C (**B**); 150 °C (**C**); and 180 °C (**D**); differences in percentage chain-length reduction among different rice starch samples: SS-120 vs. Native (**E**); SS-120 vs. SS-180 (**F**); Native: untreated rice starch; SS-120: rice starch with superheated steam at 120 °C; SS-150: rice starch with superheated steam at 150 °C; and SS-180: rice starch with superheated steam at 180 °C.

The length of amylopectin starch chains significantly affects its physicochemical properties [23] and a high proportion of short A-chains makes starch less prone to retrogradation. Table 2 and Figure 3 illustrate the distribution of different degrees of polymerization (DP) chains of rice starch: short A chains (DP 6–12), medium B1 chains (DP 13–24), long B2 chains (DP 25–36), and super long B3+ chains (DP ≥ 37) [24]. The proportion of short A chains in SS-treated starch increased from 30.70% to 37.59%, while the proportions of B1, B2, and B3+ chains decreased from 47.50% to 44.35%, from 11.40% to 10.63%, and from 10.70% to 7.42%, respectively (*p* < 0.05). It demonstrated that SS treatment could break long amylopectin starch chains into short ones [9].

Compared with native rice starch, the weight-average molecular weight (Mw) of SS-treated rice starch increased significantly (*p* < 0.05), likely due to the interaction between amylose and amylose, and between amylose and amylopectin caused by SS treatment. This finding aligns with Wang et al. [25]. The polydispersity index (PDI) was negatively correlated with the uniformity of starch molecules; a smaller PDI indicates more uniform molecular sizes. After SS-180 treatment, the PDI of rice starch decreased significantly (*p* < 0.05), indicating a more compact and uniform Mw distribution. SS-180 treatment increased the proportion of short A chains and decreased the PDI of rice starch, indicating that SS-180 treatment accelerated the depolymerization of both linear and branched starch side chains and the resolution of starch molecules, making their distribution more concentrated and stable in smaller fragments and linear molecules [26]. It demonstrated that SS-180 treatment benefited the structural modification.

### 2.5. Microstructure Analysis of Rice and Rice Flour

**Figure 4:**
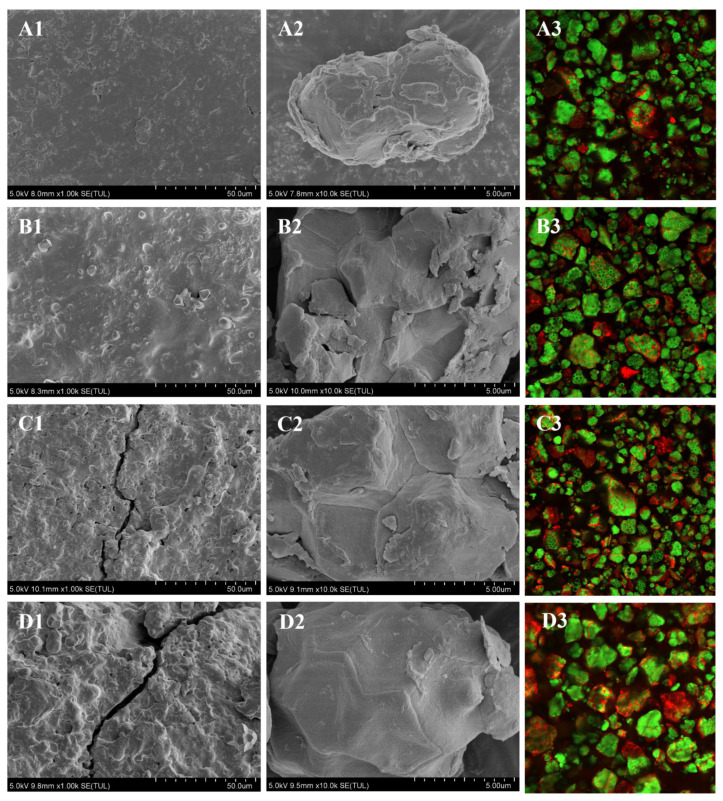
Morphological characteristics of rice flour. Script 1–2 represent rice surface and rice flour structure by scanning electron microscopy. Script 3 represents confocal laser scanning microscopy of rice flour. Untreated rice flour (**A1**–**A3**); rice flour with superheated steam at 120 °C (**B1**–**B3**); rice flour with superheated steam at 150 °C (**C1**–**C3**); and rice flour with superheated steam at 180 °C (**D1**–**D3**).

SEM micrographs (Figure 4A1–D2) revealed that native rice grains had flat surfaces, whereas SS-treated grains became rough (Figure 4B1) and had even larger cracks between starch granules and cell walls (Figure 4C1–D1). SS treatment enhanced evaporation, increasing internal cell temperature and pressure [27]. Starch granules in native rice flour were scattered, and the increase in SS treatment temperature led to aggregation and smoother surfaces of rice starch due to gelatinization (Figure 4A2–D2) [17]. Our results were consistent with Kim et al. [14], who observed similar granular changes, and Zavareze et al. [28], who noted aggregation in heat–moisture-treated rice starch.

CLSM images (Figure 4A3–D3) showed native flour with evenly dispersed proteins emitting strong green fluorescence. SS treatment caused protein and starch granule aggregation, consistent with SEM results (Figure 4). Aggregation increased with SS temperature, with SS-180 showing the most agglomerations, aligning with Wang et al.‘s findings [25]. SEM and CLSM analyses further elucidated the reasons behind the increase in average particle size of SS-150 and SS-180 rice flour (Figure 1) and the significantly higher pasting temperature of SS-treated rice flour (Figure 2).

### 2.6. Effect of SS Treatments on Rice Cake Quality

**Figure 5:**
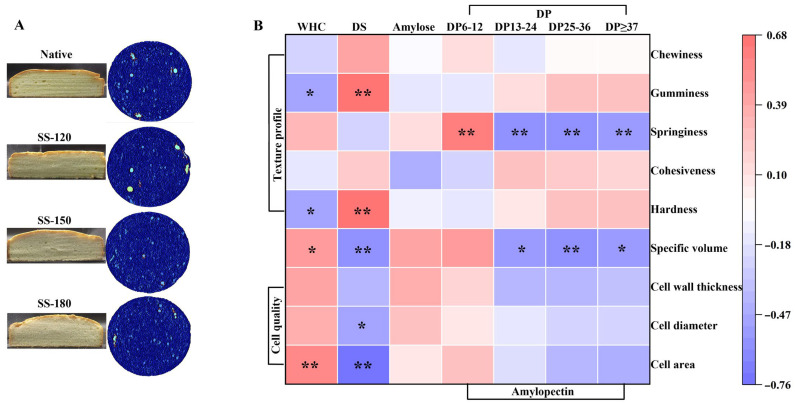
Cross-section images (**left**) and crumb structure of rice cake (**right**). (**A**). Native: untreated rice cake; SS-120: rice cake with superheated steam at 120 °C; SS-150: rice cake with superheated steam at 150 °C; and SS-180: rice cake with superheated steam at 180 °C. Association between rice cake quality and changes in water holding capacity (WHC) and starch properties (**B**). DS: damaged starch. * represents significant differences, *p* < 0.05; ** represents highly significant differences, *p* < 0.001.

High-quality rice cake is characterized by a high specific volume, a spongy and uniform crumb structure, and a soft yet elastic texture. As shown in Table 3 and Figure 5, the SS-150 and SS-180 treatments significantly improved these quality indicators compared to untreated rice flour (*p* < 0.05). The specific volumes for SS-150 and SS-180 cake reached 3.08 mL/g and 3.09 mL/g, respectively, which were attributed to the enhanced water holding capacity (Figure 1A) and the improved rheological properties of the batter (Figure 1B, Figure 2E–H). This outcome was consistent with prior findings that better batter viscosity and superior gel-forming capacity contributed to enhanced crumb structure and texture [29]. Higher pasting temperature, as observed in our study, also promotes the formation of internal cake structure [30]. In contrast, the cake made from native and SS-120 treated rice flour exhibited lower specific volumes and suboptimal textural properties, likely due to lower G′, G′′, and higher tan δ values (Figure 2F–H), indicating weaker viscoelastic networks.

A correlation analysis further elucidated the underlying mechanisms (Figure 5B). The water holding capacity was positively correlated with cell structure quality, specific volume, and springiness, and negatively correlated with hardness, gumminess, and chewiness. The increased water holding capacity of SS-treated rice flour could be linked to the expansion of starch molecules, which promoted a more orderly molecular arrangement and stabilized the internal structure of the rice cake. This structural enhancement reduced cake hardness and preserved softness during storage. On the contrary, the content of damaged starch was negatively correlated with cake cell quality, specific volume, and springiness, but positively correlated with hardness, gumminess, and chewiness. This finding was consistent with the findings of Wu et al. [31], indicating that a higher content of damaged starch leads to poorer cake quality.

Moreover, the correlation analysis revealed that the proportion of short amylopectin chains (DP 6-12) was positively associated with improved cake attributes like specific volume and springiness, while longer amylopectin chains (DP ≥ 13) had the opposite effect. SS treatment, particularly at higher temperatures, increased the proportion of short chains, which played a crucial role in reducing cake hardness and delaying retrogradation.

After 7 days of storage at 4 °C, the rice cake showed increased firmness over time. Increased storage hardness can result from starch degradation and water evaporation. Compared to native cake, the SS-180 cake showed significantly reduced hardness, increased cohesion, and improved elasticity, with delayed amylopectin degradation. Due to the reduced solubility of amylose, the increase in short A-chains and the decrease in long chains in amylopectin effectively delay the starch retrogradation of rice (Figure 1B/Table 2), SS-180 treatment significantly improved the water holding capacity of rice flour (*p* < 0.05) (Figure 1A). This improvement was beneficial for cake moisture retention during storage, preventing dryness and maintaining cake softness, which was more desirable to consumers. To sum up, short-duration, high-temperature SS treatment could greatly improve cake texture properties.

**Figure 6:**
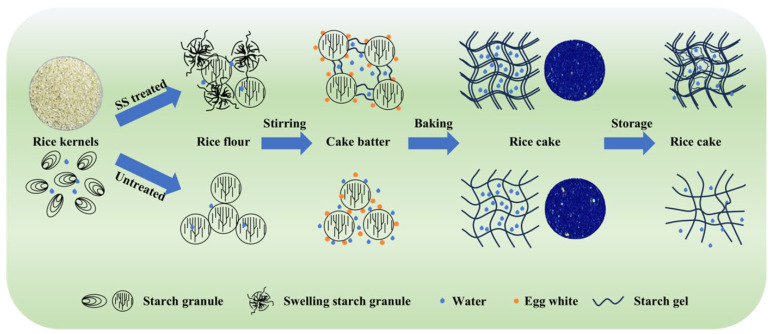
Schematic diagram of the impact of SS treatment on the quality of rice cake.

The findings are illustrated in the schematic diagram in Figure 6, which compares the effects of SS treatment on rice cake quality with the untreated one. SS treatment induced pre-gelatinization and the swelling of starch granules. When the rice flour was mixed with the egg white by stirring to form the cake batter, the pre-gelatinized starch granules promoted the internal rearrangement of starch molecules because of an easier formation of starch gel than native starch granules. It contributed to an increase in starch gel orderliness, and the egg white wrapped tightly around the starch network, improving the gas-holding capacity of the cake batter [19]. The cake batter of native rice, by contrast, formed a less cohesive system of protein, starch, and water. During baking, the SS-treated rice cake developed a more stable and orderly gel structure, enhancing the water holding capacity and creating a uniform, dense pore structure, but the gel network of native starch was not as strong. After one week of storage, the differences in the water holding capacity between the SS-treated rice cake and the untreated one were much more significant. The SS-treated rice cake exhibited a higher proportion of short amylopectin chains, which delayed starch retrogradation and helped maintain cake structure and quality. In contrast, the gel network of native starch shrank due to the lack of water holding, resulting in an uneven pore distribution of rice cake without pre-gelatinization. The starch molecules of the native rice cake rearranged, leading to retrogradation and a marked decline in cake quality, shown as a sharp increase in hardness during storage, but this process was retarded by SS-treated rice. The comprehensive analysis highlighted how SS treatment improved the processing of rice flour, ultimately improving cake quality by optimizing starch functionality and texture stability.

## 3. Materials and Methods

### 3.1. SS Treatment of Rice Kernels

Rice kernels were supplied by Yijiangqiu Grain and Oil Technology Corporation. Using SS equipment (FE-500/C, Qingdao, Shandong, China), the rice was treated at temperatures of 120 °C (SS-120), 150 °C (SS-150) and 180 °C (SS-180) for 4 min each, followed by cooling to room temperature prior to milling. The milled rice kernels were processed into rice flour using an airflow crusher (FI-11A, Beijing, China) with frequency parameters of 50 Hz, 30 Hz, and 50 Hz. The resulting rice flour was then sieved through a 200-mesh sieve and stored at 25 °C for future use.

### 3.2. Physicochemical Properties of Rice Flour

The moisture contents of native and SS-treated rice flour were measured using a rapid moisture analyzer (VM-E10, Jiangsu, China). The water holding capacity was determined by AACC International Method 56-11-02 (AACC, 2000) [32]. 

The contents of damaged starch (DS) and total starch were measured with the starch damage assay kit (K-SDAM, Megazyme International Ltd., Wicklow, Ireland) and an enzymatic kit (K-TSTA 07/11 with KOH procedure, Megazyme International Ireland Ltd., Wicklow, Ireland), respectively. Amylose content was assessed using the amylose and amylopectin assay kit (Megazyme International Ltd., Wicklow, Ireland).

Particle size distribution was determined using a particle size analyzer (Mastersizer 3000, Malvern Instruments, Worcestershire, UK) equipped with a Scirocco 3000 dry dispersion unit. In the determination of particle size distribution, D43 represents the average volume diameter, while D50 and D90 represent the diameters at which 50% and 90% of the particle volume are smaller than these values, respectively.

### 3.3. The Pasting Properties of Rice Flour

The pasting properties were analyzed using a Rapid Visco Analyzer (RVA-4, Newport Scientific Pty. Ltd., Warriewood, Australia), following the method described by Wang et al. [6]. A suspension was prepared by mixing 3.5 g of rice flour (adjusted to 14% moisture content) with 25 g of deionized water. The mixture was manually homogenized using a plastic paddle immediately before the RVA test. The analysis was conducted through a programmed heating and cooling cycle.

### 3.4. The Amylopectin Chain-Length Distribution and Weight-Average Molecular Weight of Rice Starch

The starch extraction process was conducted as follows: 500 mg of rice flour was mixed with water, followed by the addition of 10 times the amount of DMSO solution. The mixture was boiled for 2 h. After boiling, the mixture was centrifuged, and the supernatant was collected. Eight times the volume of anhydrous ethanol was then added to the supernatant. The mixture was vortexed thoroughly and left to settle at room temperature for 2 h. Subsequently, the mixture was centrifuged again, and the precipitate was collected and washed 2–3 times with distilled water. After the washing steps, the precipitate was centrifuged, and the supernatant was discarded. The remaining starch was carefully collected and dried at 40 °C. The dried starch was then ground and sieved for further analysis.

The amylopectin chain-length distribution of rice starch was analyzed using an ICS-5000 Ion Chromatography System (Thermo Fisher Scientific, Waltham, MA, USA) equipped with a Dionex™ CarboPac™ PA200 ion column (3.0 × 250 mm, 062895) [17]. The chromatographic conditions were as follows: flow rate of 0.4 mL/min, injection volume of 5 μL. The solvent system consisted of 0.2 M of NaOH and a mixture of 0.2 M of NaOH with 0.2 M of NaAc. The gradient program was as follows: 90:10 *v*/*v* at 0 min, 90:10 *v*/*v* at 10 min, 40:60 *v*/*v* at 30 min, 40:60 *v*/*v* at 50 min, 90:10 *v*/*v* at 50.1 min, and 90:10 *v*/*v* at 60 min [33].

The weight-average molecular weight of native and SS-treated rice starch was measured using a gel chromatography differential multi-angle laser light scattering system. The liquid chromatography system used was the U3000 (Thermo, Waltham, MA, USA), with the differential refractive index detector Optilab T-rEX (Wyatt Technology, Goleta, CA, USA), and the laser light scattering detector DAWN HELEOS II (Wyatt Technology, Goleta, CA, USA) with a wavelength of 663.7 nm. A gel exclusion chromatography column set with appropriate weight-average molecular weight range (Ohpak SB-805 HQ (300 × 8 mm), Ohpak SB-804 HQ (300 × 8 mm), Ohpak SB-803 HQ (300 × 8 mm)) was used. The column temperature was maintained at 60 °C, the injection volume was 200 μL, and the mobile phase consisted of 0.5% LiBr in DMSO. The flow rate was set to 0.3 mL/min with an elution gradient over 120 min. The DMSO solution has a dn/dc value of 0.07 mL/g [34].

### 3.5. Rheological Properties of Rice Flour and Rice Paste

The rheological properties of native and SS-treated rice flour and rice paste were measured using an AR1500ex rheometer (TA Instruments, New Castle, DE, USA) with a 40 mm, 1° parallel plate geometry, and a truncation gap of 1 mm. Rice flour was dispersed in distilled water (66.67% *w*/*w*) for 3 min followed by steady shear and a frequency sweep tests at a shear rate of 0.1–100 s^−1^ and an angular frequency of 0.1–100 rad·s^−1^, respectively, at 0.6% strain. Storage modulus (G’), loss modulus (G”), and loss tangent (tan δ) of native and SS-treated rice flour and rice paste were measured [35]. The rheological properties of the rice paste, the primary component for rice cake preparation, were also measured. The rice paste was composed of rice flour (30.77%), egg yolk (30.77%), milk (23.08%) and soybean oil (15.38%). The rheological analysis was performed following the same method used for rice flour, as described earlier.

### 3.6. Microstructure of Rice Flour

The microstructure of native and SS-treated rice flour was analyzed using confocal laser scanning microscopy (CLSM) (Zeiss LSM 710, Carl Zeiss MicroImaging GmbH, Jena, Germany). Fluorescein isothiocyanate (FITC) was used to stain starch green, and rhodamine B was used to stain proteins red. A 40 mg rice flour sample was dissolved in 1 mL of water, followed by the addition of 100 μL of FITC (1 mg/mL) and rhodamine B (2.5 mg/mL) for staining. The mixtures were incubated in the dark at room temperature for 1 h. A small amount of the stained sample was then placed on a glass slide and covered with a cover slip. The samples were observed under the CLSM, with fluorescein isothiocyanate (FITC) and Rhodamine B being excited at wavelengths of 488 nm and 543 nm, respectively.

Starch granule morphology was examined using SEM at an accelerating voltage of 10 kV and magnifications of 1000× or 10,000×. Samples were gold-coated prior to observation, and an acceleration voltage of 10 kV was used for imaging.

### 3.7. The Preparation of Rice Cake

The cake batter was formulated using the following ingredients: rice paste (rice flour (18.18%), egg yolk (18.18%), milk (13.64%), soybean oil (9.09%), egg white (27.27%), and sugar (13.64%). The milk and soybean oil were then added to the egg yolk and mixed thoroughly to achieve full emulsification. Rice flour was subsequently added to the mixture and blended until homogeneous [15]. The egg white and sugar were whipped at high speed for 4 min using a Royalstar mixer (Guangzhou, Guangdong, China). The whipped egg white was gradually folded into the paste in three portions, ensuring even incorporation. The batter (330 g) was then transferred to a six-inch baking mold and baked at 165 °C for 30 min in an electronic oven (S80, Hauswirt, Foshan, China) set to wind furnace mode. After baking, the cake was cooled and stored in airtight bags at 4 °C for further analysis.

### 3.8. Baking Properties of Rice Cake

The specific volume of rice cake was determined by the rapeseed displacement method, calculated as the ratio of cake volume (mL) to cake weight (g) [31].

A texture profile analysis (TPA) was conducted on fresh and 1-week-old refrigerated cake using a TA-XT2 texture analyzer, with a 35 mm circular probe. Rice cake crumb samples were cut into 25 × 25 × 25 mm pieces. An aluminum probe (36 mm diameter, P/36R) was used in the TPA model with double compression penetrating 50% of the crumb depth. Test parameters were set as follows: pre-test speed, post-test speed, test speed, and trigger force at 1 mm/s, 1 mm/s, 1 mm/s, and 2 g, respectively, with a 5 s delay between compressions [36]. Six analyses were conducted for each sample set.

Cooled rice cakes were sliced, and three 1 cm-thick slices from the cake center were selected for measuring crumb structure by C-Cell (Caliber Technology Development Co., Ltd., Beijing, China), including slice area, cell diameter, and cell wall thickness.

### 3.9. Statistical Analysis

All assays were performed in triplicate, and data were reported as mean ± standard deviation (SD). Statistical differences were evaluated using a one-way analysis of variance (ANOVA) followed by the Duncan test with SPSS Version 26.0 for Windows (SPSS Inc., Chicago, IL, USA) at a significance level of 0.05. Chart analyses and a principal component analysis (PCA) were conducted using Origin 2021 software.

## 4. Conclusions

This study has demonstrated the significant impact of SS treatment on the physicochemical properties and quality of rice flour and rice cake. The SS treatment, particularly at high temperatures, effectively modified the rice starch structure, resulting in an increased proportion of short A-chains and a more compact molecular distribution. These structural changes contribute to the improved physicochemical stability and reduced retrogradation of the starch. Moreover, SS-treated rice flour exhibited enhanced moisture retention and reduced amylase activity, extending its storage life by preventing starch hydrolysis. The high-temperature SS treatment also positively affected the milling properties, making the rice flour denser and harder at moderate temperatures, and fluffier at higher temperatures due to protein–starch aggregation and structural expansion. In the context of rice cake, the SS-180 treatment was particularly beneficial. It delayed amylopectin degradation, which helped maintain the cake’s softness and elasticity over time, while its high water holding capacity reduced starch retrogradation and prolonged shelf life. These findings suggest that SS treatment can be a valuable method for improving the textural properties and overall quality of rice-based products.

## Figures and Tables

**Table 1:** Pasting properties of rice flour.

Pasting Properties	Native	SS-120	SS-150	SS-180
Peak viscosity (Pa·s)	3044 ± 41 ^a^	2649 ± 93 ^c^	2722 ± 42 ^bc^	2767 ± 63 ^b^
Trough viscosity (Pa·s)	596 ± 22 ^a^	446 ± 17 ^b^	372 ± 35 ^c^	363 ± 33 ^c^
Breakdown (Pa·s)	2448 ± 46 ^a^	2203 ± 100 ^c^	2350 ± 34 ^b^	2404 ± 35 ^ab^
Final viscosity (Pa·s)	3202 ± 7.6 ^a^	2994 ± 177 ^b^	3221 ± 75 ^a^	3320 ± 51 ^a^
Setback (Pa·s)	2606 ± 27 ^b^	2549 ± 182 ^b^	2849 ± 81 ^a^	2957 ± 23 ^a^
Pasting time (min)	9.49 ± 0.05 ^b^	9.61 ± 0.15 ^ab^	9.68 ± 0.16 ^a^	9.73 ± 0.07 ^a^
Pasting temperature (°C)	81.31 ± 0.19 ^c^	82.11 ± 0.27 ^b^	82.65 ± 0.47 ^a^	82.72 ± 0.32 ^a^

Data were presented as mean ± SD. Values with different superscript letters in rows were significantly different, *p* < 0.05. Native: untreated rice flour; SS-120: rice flour with superheated steam at 120 °C; SS-150: rice flour with superheated steam at 150 °C; and SS-180: rice flour with superheated steam at 180 °C.

**Table 2:** Amylopectin chain-length distribution and weight-average molecular weight of rice starch.

	Native	SS-120	SS-150	SS-180
DP6-12 (%)	30.40 ± 0.01 ^c^	31.75 ± 0.11 ^b^	31.67 ± 0.05 ^b^	37.59 ± 0.12 ^a^
DP13-24 (%)	47.50 ± 0.11 ^a^	46.25 ± 0.21 ^b^	46.81 ± 0.05 ^c^	44.35 ± 0.16 ^d^
DP25-36 (%)	11.40 ± 0.02 ^a^	11.27 ± 0.06 ^b^	11.22 ± 0.01 ^b^	10.63 ± 0.05 ^c^
DP ≥ 37 (%)	10.70 ± 0.09 ^a^	10.73 ± 0.10 ^a^	10.31 ± 0.02 ^b^	7.42 ± 0.08 ^c^
Mw (kDa)	88,127.87 ± 5299.20 ^c^	127,439.13 ± 1406.56 ^b^	142,088.03 ± 5482.78 ^a^	122,115.39 ± 8838.54 ^b^
PDI	2.88 ± 0.26 ^a^	2.88 ± 0.18 ^a^	2.71 ± 0.09 ^a^	2.28 ± 0.29 ^b^

DP: degree of polymerization; Mw: weight-average molecular weights; PDI: polydispersity index; Data were presented as mean ± SD. Values with different superscript letters in rows were significantly different, *p* < 0.05. Native: untreated rice starch; SS-120: rice starch with superheated steam at 120 °C; SS-150: rice starch with superheated steam at 150 °C; and SS-180: rice starch with superheated steam at 180 °C.

**Table 3:** Baking properties of rice cake.

Time	Crumb Structure	Native	SS-120	SS-150	SS-180
0 day	Cell area (%)	50.57 ± 0.25 ^bc^	50.15 ± 0.73 ^c^	51.27 ± 0.21 ^a^	51.18 ± 0.29 ^a^
0 day	Cell diameter (mm)	1.71 ± 0.04 ^b^	1.73 ± 0.11 ^ab^	1.83 ± 0.00 ^a^	1.79 ± 0.07 ^ab^
0 day	Cell wall thickness (mm)	0.42 ± 0.01 ^a^	0.43 ± 0.01 ^a^	0.44 ± 0.01 ^a^	0.44 ± 0.01 ^a^
0 day	Specific volume (mL/g)	2.86 ± 0.08 ^b^	2.96 ± 0.06 ^ab^	3.08 ± 0.05 ^a^	3.09 ± 0.09 ^a^
0 day	Hardness (N)	1.81 ± 0.07 ^b^	2.03 ± 0.15 ^a^	1.30 ± 0.29 ^d^	1.59 ± 0.13 ^c^
0 day	Cohesiveness	0.73 ± 0.02 ^a^	0.71 ± 0.02 ^b^	0.70 ± 0.01 ^b^	0.71 ± 0.02 ^b^
0 day	Springiness (mm)	7.33 ± 0.25 ^b^	7.64 ± 0.24 ^ab^	6.92 ± 1.36 ^b^	8.51 ± 0.41 ^a^
0 day	Gumminess (N)	1.32 ± 0.05 ^a^	1.44 ± 0.12 ^a^	0.90 ± 0.20 ^c^	1.11 ± 0.10 ^b^
0 day	Chewiness (mJ)	9.65 ± 0.50 ^a^	10.98 ± 1.19 ^a^	6.43 ± 2.52 ^c^	9.51 ± 1.28 ^a^
7 day	Hardness (N)	6.05 ± 0.51 ^a^	5.77 ± 0.14 ^ab^	5.83 ± 0.21 ^ab^	5.30 ± 0.30 ^b^
7 day	Cohesiveness	0.60 ± 0.06 ^b^	0.60 ± 0.03 ^b^	0.59 ± 0.01 ^b^	0.71 ± 0.07 ^a^
7 day	Springiness (mm)	9.20 ± 0.10 ^b^	9.52 ± 0.06 ^a^	9.49 ± 0.14 ^a^	9.14 ± 0.14 ^b^
7 day	Gumminess (N)	3.65 ± 0.61 ^a^	3.47 ± 0.19 ^a^	3.44 ± 0.15 ^a^	3.77 ± 0.16 ^a^
7 day	Chewiness (mJ)	34.70 ± 5.06 ^a^	33.08 ± 1.99 ^a^	32.66 ± 1.12 ^a^	34.40 ± 1.00 ^a^

Data were presented as mean ± SD. Values with different superscript letters in rows were significantly different, *p* < 0.05. Native: untreated rice cake; SS-120: rice cake with superheated steam at 120 °C; SS-150: rice cake with superheated steam at 150 °C; and SS-180: rice cake with superheated steam at 180 °C.

## Data Availability

Data are contained within this article.
